# Effects of Systolic Blood Pressure on Brain Integrity in Multiple Sclerosis

**DOI:** 10.3389/fneur.2018.00487

**Published:** 2018-06-25

**Authors:** Daiana E. Dossi, Hernán Chaves, Evelyn S. Heck, Sofía Rodriguez Murúa, Fernando Ventrice, Rohit Bakshi, Francisco J. Quintana, Jorge Correale, Mauricio F. Farez

**Affiliations:** ^1^Department of Neurology, Raúl Carrea Institute for Neurological Research (FLENI), Buenos Aires, Argentina; ^2^Department of Diagnostic Imaging, Raúl Carrea Institute for Neurological Research (FLENI), Buenos Aires, Argentina; ^3^Center for Research on Neuroimmunological Diseases (CIEN), Raúl Carrea Institute for Neurological Research (FLENI), Buenos Aires, Argentina; ^4^Department of Neurology, Partners Multiple Sclerosis Center, Brigham and Women's Hospital, Harvard Medical School, Boston, MA, United States; ^5^Laboratory for Neuroimaging Research, Departments of Neurology and Radiology, Brigham and Women's Hospital, Boston, MA, United States; ^6^Ann Romney Center for Neurologic Diseases, Department of Neurology, Brigham and Women's Hospital, Harvard Medical School, Boston, MA, United States; ^7^The Broad Institute of MIT and Harvard, Cambridge, MA, United States

**Keywords:** hypertension, systolic blood pressure, multiple sclerosis, white-matter integrity, fractional anisotrophy

## Abstract

**Background:** In MS patients, hypertension is associated with a delayed diagnosis and an increased risk of progression. Understanding the mechanisms of this association could potentially lead to improved prevention of disease progression. We aimed to establish whether high blood pressure contributes to white-matter injury and brain atrophy in MS.

**Methods:** Cross-sectional study of 95 patients with RRMS. Estimates of fractional anisotropy, gray-matter volume and lesion load were obtained from 3T MRI. We used fractional anisotropy voxel-based statistics to establish the effect of blood pressure on white matter tracts. Additionally, we used voxel-based morphometry (VBM) to study the effect on gray matter integrity.

**Results:** Only 29.5% had normal blood pressure levels, with 52.6% suffering from prehypertension and 17.9% with hypertension. Increasing systolic blood pressure was associated with damage to posterior white-matter tracts as well as greater levels of gray matter atrophy, in particular in the frontal cortex. Age-adjusted linear regression indicated that neither lesion volume (β = 0.002, 95%CI: 0.02–0.02; *p* = 0.85) or lesion number (β = −0.004, 95%CI: 0.03–0.02; *p* = 0.74) were associated with systolic blood pressure.

**Conclusions:** Prehypertension and hypertension are frequent in MS. Increased blood pressure is related to white- and gray-matter integrity, both related to MS disability outcomes. These findings suggest attention to the control of blood pressure in MS patients.

## Introduction

Multiple sclerosis (MS) is a chronic autoimmune disease characterized by the destruction of myelin and other antigens in the central nervous system (CNS) (1). Brain, spinal cord, and optic nerve lesions arise from the infiltration of autoreactive mononuclear peripheral blood cells (1). Lesions are typically located in the white matter (WM) of periventricular, juxtacortical, infratentorial, and spinal cord areas of the CNS (2). Additionally, gray matter (GM) lesions and brain atrophy are observed commonly during the course of the disease (3). Brain atrophy reflects tissue loss and represents a global measure of both severe demyelination and axonal loss in MS (3). The pathogenesis and course of the disease are thought to result from complex interactions between genetic and environmental factors (4, 5). Of note, substantial heterogeneity exists in disease severity and several comorbidities have been proposed that may account for some of the observed differences in disease course among individuals (6).

Recently, epidemiological findings suggest a relationship between MS and several cardiovascular comorbidities, including obesity (7), insulin resistance (7, 8), dyslipidemia (9), and hypertension (8, 10); the latter being the most frequent cardiovascular comorbidity reported in MS (11). In chronic medical conditions, cardiovascular comorbidities are associated with decreased quality of life and increased mortality (11). In MS in particular, these comorbidities are associated with a delayed diagnosis, worse magnetic resonance imaging (MRI) outcomes (12), and increased risk of disease progression (13). For example, a direct relationship between cardiovascular risk factors and clinical status as measured by the Expanded Disability Status Scale (EDSS) has been reported (14). Furthermore, higher low-density lipoprotein (LDL) cholesterol and total cholesterol levels were associated with inflammatory MRI measures (9). Additionally, a large MS cohort study reported that hypertension and heart disease were associated with brain atrophy and that obesity, was associated with T1 lesion volumes (12). Finally, we recently reported that a high sodium intake, a regulating factor of blood pressure (BP), is linked to MS disease activity (15). Understanding the distribution and mechanisms of cardiovascular comorbidities in MS could then potentially lead to better management of patients and improved diagnosis and prevention of disease progression.

Despite the strong epidemiological evidence, there is a paucity of studies exploring pathophysiological mechanisms to explain the association between MS and cardiovascular comorbidities. In particular, the mechanisms linking MS severity and elevated BP are poorly understood. Hypertension is associated with both cerebrovascular and cardiovascular diseases and is the greatest risk factor for mortality in those conditions (16). Moreover, elevated systolic BP has been previously linked to brain atrophy, WM injury and increased blood-brain barrier permeability in a range of conditions other than MS (17–19).

We thus hypothesize that elevated BP contributes to MS progression by affecting WM and GM integrity. To address this question, we evaluated the relationship between BP and a sensitive brain MRI measure of structural WM change (fractional anisotropy-FA) in a cross-sectional cohort of patients with MS. We additionally tested for an association between elevated BP and MS cerebral lesion load as well as GM atrophy. Establishing whether BP contributes to structural brain changes in MS would highlight the potential relevance of aggressive screening and management of hypertension.

## Methods

### Study design and patients

This study was carried out in accordance with the recommendations of the Ethics guidelines of the Raúl Carrea Institute for Neurological Research Ethics Committee. The protocol was approved by the Raúl Carrea Institute for Neurological Research Ethics Committee. All subjects gave written informed consent in accordance with the Declaration of Helsinki. One hundred consecutive patients with RRMS according to the 2010 International Panel criteria (2) who were seen at the MS clinic of the Raúl Carrea Institute for Neurological Research were recruited from November 2013 to July 2015. Patients needed to be 18 years old or older to be included. We excluded pregnant women and patients with a previous history of other neurological disorders that may affect MRI assessment. We assessed the patient's current medications, with particular attention to antihypertensive agents.

### Blood pressure measurement

Blood pressure was measured in one session at the time of enrollment. Classification of BP for adults was based on the 7th Report of the Joint National Committee on prevention, detection, evaluation, and treatment of high BP (16). Categories were as follows: Normal (systolic-SBP < 120 and diastolic-DBP < 80), Prehypertension (SBP 120-139 or DBP 80-89), Stage 1 hypertension (SBP 140-159 or DBP 90-99) and Stage 2 hypertension (SBP ≥ 160 or ≥ 100). Due to our patients' age range we expected none or few patients in Stage 2 hypertension; therefore, we used a pooled hypertension group by collapsing Stage 1 and 2. After the finalization and image analysis of our study, a new set of recommendations were published: the 8th Report of the Joint National Committee on prevention, detection, evaluation, and treatment of high BP (20) and later the 2017 ACC/AHA/AAPA/ABC/ACPM/AGS/APhA/ASH/ASPC/NMA/PCNA Guideline for the Prevention, Detection, Evaluation, and Management of High Blood Pressure in Adults (21). In this guideline, BP was categorized as normal (< 120/80 mm Hg), elevated (120–129/ < 80 mm Hg), stage 1 hypertension (130–139/80–89 mm Hg), or stage 2 hypertension (≥140/90 mm Hg). Since the majority of the analysis were done with lineal uncategorized values of BP, the update on guidelines definition did not modify our original analysis.

For BP recordings, patients were requested to sit in an empty office for at least 5 min in a chair with their feet on the floor and arm supported at the level of the heart. BP measurement was delayed if the patient smoked, consumed caffeine, or practiced exercise within 30 min of measurement. The BP measurement was made by trained non-medical personnel to avoid the “white coat effect.” An automatic BP monitor was used (Microlife WatchBP Office Blood Pressure Monitor, Microlife, Switzerland) following these steps: first, the dominant arm was identified, then three measurements separated by 60 s intervals were made and the average of those results were considered as the final value.

### Image acquisition and analysis

Brain MRI images were acquired on a Signa HDxt 3T scanner (General Electric, Milwaukee, IL) using an 8-channel head coil. Both 3-dimensional (3D) T1-weighted and FLAIR pulse sequences were used to measure lesion load and brain volumes. We acquired a sagittal 3D fast spoiled gradient-echo T1-weighted sequence (512 × 512 matrix; field of view = 25 cm; slice thickness = 1.1 mm; TR = 7,400 ms; TE = 2,400; TI = 450 ms; flip angle = 15°) and isotropic 3D FLAIR (512 × 512 matrix; field of view = 25 cm; slice thickness = 1 mm; TR = 8,200 ms; TE = 136 ms; TI = 2,200 ms).

Lesions were segmented by the fully automated lesion growth algorithm (22) as implemented in the LST toolbox version 2.0.15 (https://www.applied-statistics.de/lst.html) for SPM12. This algorithm is able to segment T2-hyperintense lesions from a combination of T1 and FLAIR images. It first segments the T1 image into the three main tissue classes [cerebrospinal fluid (CSF), GM, and WM]. This information is then combined with the FLAIR intensities in order to calculate lesion maps. Using this method, lesion number and volumes was estimated.

In addition to this, estimated lesion masks were then automatically filled using an internal filling method where candidate region voxels where replaced by random intensities from a Gaussian distribution generated from the normal-appearing WM intensities and then filtered to reintroduce the original spatial variation in WM (23). We filled MS lesions in order to increase the accuracy of brain volumes estimates as previously described (24).

For brain volumes, we processed the T1-weighted images using the CAT12 toolbox (http://www.neuro.uni-jena.de) in SPM12 (http://www.fil.ion.ucl.ac.uk/spm) running MATLAB 8.5.0. Brain parenchymal fraction, WM fraction, and GM fraction were calculated as previously described (25). These global volumes were used for descriptive purposes and not considered as main predictor variables.

We acquired diffusion tensor imaging (DTI) data using the following parameters: 55 gradient directions, *b*-value 1,000 s/mm^2^, 256 × 256 matrix; field of view = 24 cm; slice thickness = 3.5 mm; in plane voxel size (pixel size) = 0.71 × 0.78 mm, TR = 10,000 ms; TE = 88.7 ms. To estimate the impact of BP in WM integrity, we performed FA voxel-based statistics using the ACID Toolbox written in MATLAB (version R2016b, Mathworks, USA), as previously described (26). DTI data were visually inspected and found to be free of artifacts using the residual error map of the tensor fit to detect outliers. The DTI data were corrected for motion and eddy current effects using ECMOCO from the ACID Toolbox. All resulting maps were of good quality. FA values were generated from the pre-processed DTI data. The default settings of the SPM12 normalization software were used for each registration approach. The b0 image was first co-registered using an affine transformation to the standard SPM12 EPI template. The same transformation was subsequently applied to the corresponding FA image and to the DW images. Finally, images were smoothed prior to model specification and analysis.

To estimate differences in regional brain volume according to BP, a voxel-based morphometry (VBM) approach was used (27), a technique previously used to estimate WM and GM atrophy in MS patients (28, 29). First, lesion-filled 3D T1-weighted images were segmented in GM, WM and CSF, as described above. Then, GM and WM segmented images of all subjects, were used to produce GM and WM templates and drive the deformation to the templates. At each iteration, the deformations, calculated using the DARTEL registration method (30), were applied to GM and WM, with an increasingly good alignment of subject morphology, to produce templates. Spatially normalized images were then modulated to ensure that the overall amount of each tissue class was not altered by the spatial normalization procedure. To better align the final template with the Montreal Neurologic Institute (MNI) space, an affine registration between the customized GM template and the statistical parametric mapping GM template (in the MNI space) was also calculated. The same transformation was applied to the WM customized template. The images were then smoothed with an 8 mm full width at half maximum (FWHM) Gaussian kernel. Finally, we overlaid the T maps with the probabilistic fiber map included in SPM12 to provide a *post-hoc* description of the area of the GM significant voxels.

### Statistical analysis

We aimed to establish if systolic BP (SBP) was associated with increased WM injury (as measured by FA, primary outcome). Our secondary outcomes were to assess if SBP was related to lesion load and regional GM volumes. For our primary outcomes, we used FA measures in a linear model as the dependent variable and the following covariates: SBP, age, smoking status (smoker, non-smoker), gender, hypertension treatment (treatment vs. no treatment), MS disease-modifying treatment, vitamin D level (continuous). The T maps obtained for the covariates were assessed for significance with threshold-free cluster enhancement with an α of 0.001.

To achieve our secondary analysis, we used a linear regression with log-transformed WM lesion volume as the dependent variable and continuous SBP as the independent variable, adjusting for the same co-variates as above. In exploratory analysis, only systolic BP (our variable of interest) and age demonstrated significance to be included in our model, and all other variables were discarded after assessing for confounding and effect modification. The same analysis was also carried out for lesion number. For GM volumes, a linear model was generated after VBM and T maps were analyzed as mentioned above.

*P* < 0.05 were considered significant. Unless otherwise noted, mean ± standard deviation is reported. All statistical procedures were performed with Stata version 12.1 (Statacorp).

## Results

### Prevalence of elevated blood pressure

One hundred patients were initially recruited in our study. Two patients later refused BP recordings and 3 patients cancelled MRI. Thus, 95 patients were finally included, 57 of whom were female (60%). Patients were aged 19-66 (median 32, IQR 32-45). Regarding BP, 28 (29%) were normal, 50 (53%) had pre-hypertension, and 17 (18%) had hypertension. According to the 2017 guidelines, 29% were normal, 18% had elevated BP, and 53% were hypertense. Patient characteristics are shown in Table 1 (including a breakdown according to BP levels).

**Table 1:** Characteristics of study participants.

	**All patients (*n* = 95)**	**Normotensive (*n* = 28)**	**Prehypertensive (*n* = 50)**	**Hypertensive (*n* = 17)**	***P-*value**
Female, *n* (%)	57 (60)	23 (82)	28 (56)	6 (35)	0.006
Age, median (range)	37 (19–66)	36.5 (19–53)	37.5 (19–66)	41 (29–58)	0.1
EDSS, median (range)	0 (0–4.5)	0 (0–4.5)	0 (0–4.5)	0 (0–3.5)	0.5
Disease duration, median years (range)	7.5 (0–20)	5 (0–20)	9 (0–18)	5.5 (0–14)	0.16
Age at diagnosis, median (range)	31 (17–52)	29.5 (17–43)	30.5 (17–52)	35.5 (18–47)	0.23
Treatment, n (%)					0.09
*Untreated*	3 (3)	0 (0)	3 (6)	0 (0)	
*IFN*	40 (43)	14 (50)	15 (31)	11 (65)	
*Glatiramer acetate*	12 (13)	3 (11)	9 (18)	0 (0)	
*Fingolimod*	29 (31)	9 (32)	14 (29)	6 (35)	
*Natalizumab*	4 (4)	2 (7)	2 (4)	0 (0)	
*Other*	7 (7)	0 (0)	7 (14)	0 (0)	
Systolic blood pressure (mmHg)	125 (13)	112 (5)	126 (7)	144 (7)	< 0.0001
Diastolic blood pressure (mmHg)	81 (12)	70 (6)	81 (6)	98 (11)	< 0.0001
Current smoking	9 (9)	3 (11)	3 (6)	3 (18)	0.3
Hypertension treatment	8 (8)	0 (0)	6 (12)	2 (12)	0.1
Brain T2 lesion number, median (range)	21 (1–91)	17 (1–58)	20 (1–91)	26 (6–51)	0.7
Brain T2 lesion volume (cm^3^)	3.6 (0.03–56.21)	4.4 (0.03–30.5)	3.4 (0.14–56.2)	3.27 (0.7–17.6)	0.99

### Elevated blood pressure and white-matter integrity

**Figure 1:**
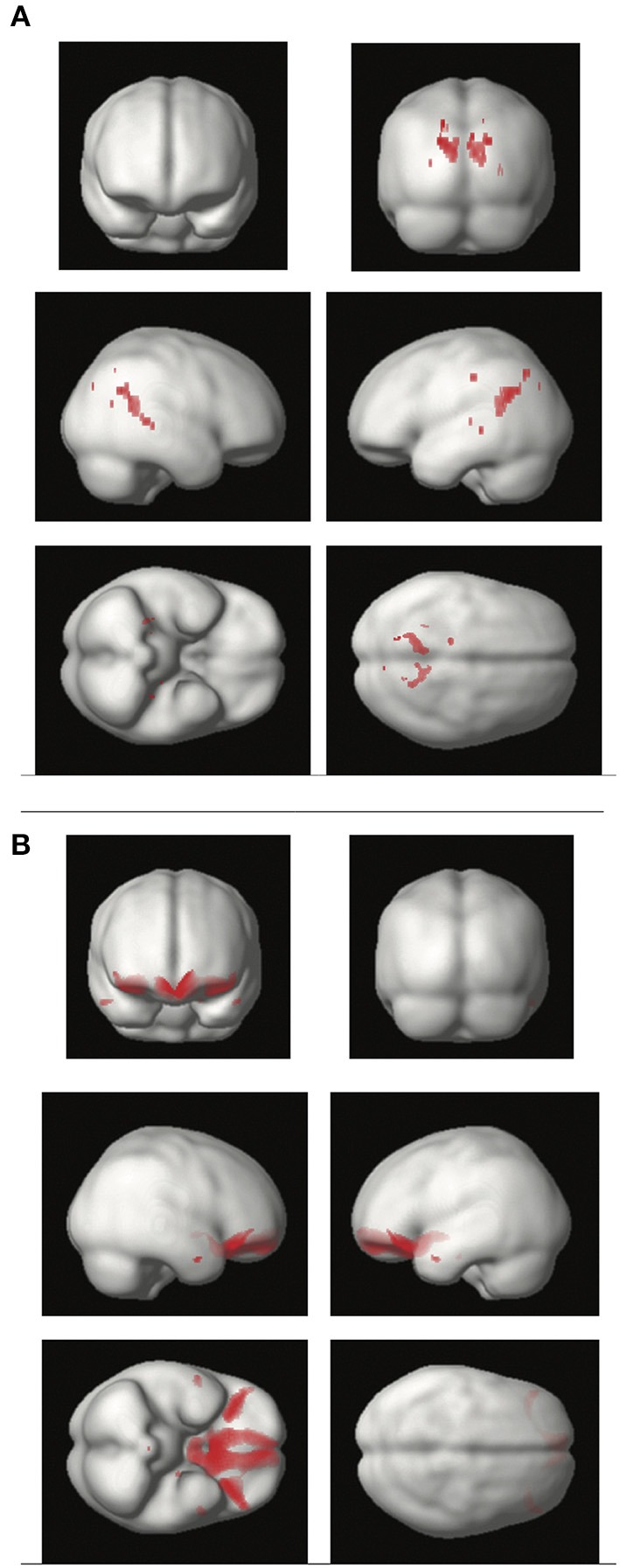
Regions of the cerebral white matter **(A)** and gray matter **(B)** in which systolic blood pressure is inversely associated with tissue integrity. **(A)** Brain render depicting white matter areas associated with decreased fractional anisotropy. Voxel-based regression included fractional anisotropy as the dependent variable and systolic blood pressure as the independent variable. Age, gender, and smoking were added as covariates. **(B)** Brain render showing gray matter areas with reduced volume associated with increasing systolic blood pressure. Voxel-based morphometry included gray matter as the dependent variable, with systolic blood pressure as the independent variable. Age, gender, smoking, and total intracranial volume were covariates.

To establish the impact of SBP on WM integrity, we performed a voxel-based regression analysis with measures of FA as the dependent variables and SBP as the main predictor. As shown in Figure 1 and Table 2, SBP was independently associated with bilateral decreased FA in voxels located in the precuneus, and middle and posterior cingulate gyrus (Figure 1 and Table 2). Thus, high levels of SBP were associated with reduced integrity of WM.

**Table 2:** Brain regions exhibiting fractional anisotropy and voxel based morphometry abnormalities.

**Area**	**Side**	***T* score**	***P* value**
**DECREASED FRACTIONAL ANISOTROPY**			
Precuneus	Left	3.6	0.001
Precuneus	Right	2.9	0.002
Middle cingulate gyrus	Left	3.4	0.001
Middle cingulate gyrus	Right	2.6	0.006
Posterior cingulate gyrus	Left	2.4	0.009
Posterior cingulate gyrus	Right	2.8	0.003
**DECREASED GRAY MATTER VOLUME**			
Posterior orbital gyrus	Left	3.53	0.001
Posterior orbital gyrus	Right	3.05	0.002
Medial frontal cortex	Left	3.16	0.001
Medial frontal cortex	Right	3.04	0.002
Subcallosal area	Left	2.95	0.002
Subcallosal area	Right	2.86	0.003

### Elevated blood pressure and brain atrophy

As shown in Figure 1 and Table 2, SBP was inversely associated with regional VBM-derived GM volumes bilaterally in the posterior orbital gyrus, the medial frontal cortex and the subcallosal area. Thus, SBP was associated with brain atrophy in frontal areas.

### Relationship between white-matter areas affected by SBP and disability

To test whether WM areas found to be affected by SBP had an impact in disability, we performed an ordinal regression analysis with EDSS score as the dependent variable and measures of FA as the predictors after adjustment by age, gender and smoking. We found a positive correlation between FA values in the left and right cingulate gyrus (*P* = 0.04 and *P* = 0.013, respectively). We did not detect a significant association between areas in which greater atrophy linked to higher BP and EDSS. Thus, in our cohort, only WM areas linked to SBP contributed to MS disability as measured by EDSS.

### Blood pressure and lesion size or number

Elevated BP can have a significant impact on WM damage and is associated with the appearance of WM hyperintensities in locations similar to MS (17). Thus, we hypothesized that elevated BP may contribute to WM lesion size or number.

We built a linear model with lesion volume as the dependent variable and age and systolic BP as the predictors. Age-adjusted linear regression indicated that neither lesion volume (β = 0.002, 95% CI: −0.02 to 0.02; *p* = 0.85) nor lesion number (β = −0.004, 95% CI: −0.03 to 0.02; *p* = 0.74) was associated with systolic BP. Thus, BP was not related to overt MS lesions.

## Discussion

In patients with RRMS, increasing SBP was associated with WM injury and greater brain atrophy. Although no direct comparison to healthy controls was made, our results indicate that elevated SBP is frequent in MS. Moreover, damage in some areas of WM affected by SBP correlated with higher physical disability on the EDSS.

Extensive work has been done in healthy controls to establish the impact of SBP and other vascular factors in brain aging (17, 31). In those studies, a clear association between SBP and WM injury and brain atrophy has been established. Although, initially, the findings referred to a link between SBP and WM hyperintensities, damage was later linked to more subtle measures of WM integrity such as FA, even in mid-life individuals, such as the cohort included in our study (17). Our work shows that raised SBP contributes to WM damage and brain atrophy in young and mid-life MS patients. A recent MS cohort study addressing the impact of cardiovascular factors (12), did not detected a significant impact in WM by hypertension, but found a similar impact on cortical atrophy. This discrepancy on WM results might be explained by the more sensitive (FA) method used in our study, of particular relevance in young cohorts.

A wide range of biological factors are thought to contribute to clinical and MRI outcomes in patients with MS, creating a complex disease pathophysiology (1). Previous studies show that high sodium intake is associated with greater clinical and MRI activity in both MS animal models and in patients with the disease (15, 32). The mechanisms of this association remain largely unknown. High sodium intake is also associated with hypertension, which, in turn, is one of the leading causes of cerebrovascular disease and mortality worldwide (16). Thus, in addition to the immunological mechanisms previously described linking sodium to MS pathophysiology (32, 33), high sodium intake could also affect MS severity indirectly through increases in SBP. One can consider possible direct mechanisms explaining the relationship between elevated BP and MS such as subtle myelin injury and increased blood brain barrier (BBB) permeability. Microvascular damage due to hypertension can increase arterial stiffness and generate decreased oxygen loads, with subsequent chronic myelin injury (34). This mechanism however does not explain the differential affection of posterior WM tracts and anterior GM. Of interest, although other tractography and VBM studies in MS patients have found similar areas affected (28), studies in healthy controls show that SBP impacts more on anterior WM tracts. This may suggest a differential interaction between SBP and autoimmunity. The second potential mechanism relates to increases in BBB permeability due to elevated SBP. Previous reports in animal models suggest that raises in SBP increase BBB leakage, and may thus facilitate the entry of immune cells and other inflammatory mediators into the brain parenchyma (19). In addition to this, Angiotensin II, a key component of the renin-angiotensin system (RAS) can also enter the brain during hypertension and influence the immune system through the activation of Angiotensin type 1 receptor (AT1R) in astrocytes and microglia (35), of great relevance to MS progression (36). A final potential mechanism could be that hypertension leads to small vessel occlusive disease and therefore to hypoperfusion and subtle brain damage (37). This fails to explain, however, the lack of association between SBP and lesion load, but could account for additional chronic damage to the brain relevant to MS progression.

Regardless of the potential mechanisms, comorbidities are an increasing concern for MS practitioners. Recently, an international workshop on comorbidities in MS was held (6). In a meta-analysis, the panel identified hypertension as one of the most frequent comorbidities in MS. In line with previous reports, they highlighted that hypertension, as well as other comorbidities, may be associated with worse outcomes. Moreover, they generated recommendations for future research, including exploring the mechanisms of the effects of comorbidity on MS as a means of identifying potential approaches to mitigating their impact. Our study goes along with this recommendation by identifying structural correlates of the effect of hypertension on cerebral MS related pathology, raising the possibility that elevated BP may contribute to disease progression.

There are several limitations of our study worthy of comment. First, our sample consisted of a cohort of white Hispanics from a single center, and may not be generalizable to the worldwide MS population. Second, although every consecutive patient was invited, participation was voluntary, and healthier, less disabled patients were more likely to accept invitation. This selection bias may have been negligible due to the relative young age and low disability of our cohort. Third, we did not include a comparison healthy control cohort, which may have shed some more information regarding the differential affection in MS and healthy controls. Further studies are required to compare both groups. The estimated prevalence in Argentina of prehypertension and hypertension is 35 and 12.2%, respectively, for a similar age group (38). Therefore, it appears that hypertension is more prevalent in MS. However, because blood pressure and hypertension were measured and defined differently, we were very cautious to avoid any misleading comparison. Fourth, we studied our patients in a cross-sectional fashion; longitudinal studies are required to further elucidate the long-term impact of BP on clinical and MRI-defined MS progression. Despite these limitations, our study has a clear impact: there is an unmet need to address the early detection and treatment of hypertension in patients with MS. Furthermore, additional research is warranted to fully characterize the role of hypertension in contributing to the pathophysiology of MS.

In conclusion, our study shows that prehypertension and hypertension are common among patients with MS. Moreover, elevated BP is associated with reduced brain integrity. Since WM and GM integrity are associated with disability outcomes during disease progression, it is of importance to avoid additional factors injuring the brain. These findings further suggest the importance of aggressive, early management of hypertension as a preventive strategy, not only for cardiovascular and cerebrovascular events, but also potentially, to limit MS disease progression. Our results, suggests that MS practitioners should measure and aim for optimum control of BP.

## Disclosures

RB has received consulting fees from EMD Serono, Genentech, Sanofi-Genzyme, and Novartis and research support from Biogen, EMD-Serono, Novartis, and Sanofi-Genzyme. FQ serves on the editorial board for Systems Biomedicine, Inmunologia, American Journal of Clinical and Experimental Immunology, is an associate editor for Immunology (UK), is an advisory board member for Seminars in Immunopathology; received research support from Harvard Medical School, BADERC, NMSS. JC is a board member of Merck-Serono Argentina,Novartis Argentina,Genzyme LATAM,Genzyme global, Biogen-Idec LATAM, and Merck-Serono LATAM. JC has received reimbursement for developing educational presentations for Merck-Serono Argentina, Merck- Serono LATAM, Biogen-Idec Argentina, Genzyme Argentina, Novartis Argentina, Novartis LATAM, Novartis Global, and TEVA Argentina as well as professional travel/accommodations stipends. MF has received travel accommodations from Teva, Merck-Serono, Biogen-Idec and Novartis. Dr. Farez has also received research funds from Biogen-Idec and Novartis Argentina.

## Author contributions

DD, HC, EH, SR, and FV: acquisition of data, analysis, and interpretation. RB, FQ, and JC: critical revision of the manuscript for important intellectual content. MF: study concept and design, data analysis and interpretation, manuscript writing, study supervision.

### Conflict of interest statement

The authors declare that the research was conducted in the absence of any commercial or financial relationships that could be construed as a potential conflict of interest.
